# Perception-Action Coupling Target Tracking Control for a Snake Robot via Reinforcement Learning

**DOI:** 10.3389/fnbot.2020.591128

**Published:** 2020-10-20

**Authors:** Zhenshan Bing, Christian Lemke, Fabric O. Morin, Zhuangyi Jiang, Long Cheng, Kai Huang, Alois Knoll

**Affiliations:** ^1^Department of Informatics, Technical University of Munich, Munich, Germany; ^2^Department of Informatics, Ludwig Maximilian University of Munich, Munich, Germany; ^3^School of Data and Computer Science, Sun Yat-sen University, Guangzhou, China

**Keywords:** snake robot, target tracking, reinforcement learning, motion planning, visual perception

## Abstract

Visual-guided locomotion for snake-like robots is a challenging task, since it involves not only the complex body undulation with many joints, but also a joint pipeline that connects the vision and the locomotion. Meanwhile, it is usually difficult to jointly coordinate these two separate sub-tasks as this requires time-consuming and trial-and-error tuning. In this paper, we introduce a novel approach for solving target tracking tasks for a snake-like robot as a whole using a model-free reinforcement learning (RL) algorithm. This RL-based controller directly maps the visual observations to the joint positions of the snake-like robot in an end-to-end fashion instead of dividing the process into a series of sub-tasks. With a novel customized reward function, our RL controller is trained in a dynamically changing track scenario. The controller is evaluated in four different tracking scenarios and the results show excellent adaptive locomotion ability to the unpredictable behavior of the target. Meanwhile, the results also prove that the RL-based controller outperforms the traditional model-based controller in terms of tracking accuracy.

## 1. Introduction

Inspired by real snakes, snake-like robots are designed as a class of hyper-redundant mechanisms in order to achieve the agility and adaptability of their biological counterparts. Their long and narrow bodies with many degrees of freedom (DOF) enable them to perform diverse tasks that could never be carried out by other kinds of mobile robots, such as search and rescue in disaster scenes (Evan, 2017), complex teleoperation in space (Walker and Hannan, 1999), and even minimally invasive cardiac surgery (Webster et al., 2006). However, this high level of flexibility also corresponds to a complex control task involving the internal regulation of body joints and external interaction with the ground, in which model-based methods usually fail to control the robots adaptively in a dynamically changing environment.

Vision-guided locomotion, as one of the essential skills for moving in changeable scenarios, is a must-have capability for snake-like robots, to ensure that they can be deployed in an unattended environment by human operators. With the help of the visual information, the snake-like robots can solve more complex and realistic tasks, such as target tracking and obstacle avoidance. Especially in field operations, such as disaster rescue tasks and surveillance tasks, the target tracking capability can greatly improve the performances of the snake-like robots. Yet this kind of locomotion control still remains a challenging task for snake-like robots, since it involves not only the locomotion but also the target information obtained by cameras. For controlling the locomotion, different types of methods have been studied including the sinusoid-based methods (Hirose, 1993), CPG-based methods (Bing et al., 2017), and the dynamics-based methods (Miller, 1988). However, none of these methods can be used directly to perform vision-based tracking tasks. Such tracking tasks require the robot to be agile and adapt to their target trajectories with unpredictable changes in velocity or direction of travel, which is extremely challenging for traditional model-based methods. Our proposal is completely different as it tackles the object tracking and camera control pipeline in an end-to-end manner, based on reinforcement learning.

Strategies based on Reinforcement Learning (RL) are promising solutions for performing target tracking for a snake-like robot. This is because a RL-trained controller can take the visual image directly as the input, while simultaneously fully exploring the locomotion capabilities compared with model-based methods. This is particularly suitable for snake-like robots with redundant degrees of freedom. Although RL-based methods have been adopted to control mobile vehicle platforms (Morbidi and Mariottini, 2013; Yun et al., 2018; Luo et al., 2019), the effectiveness of such methods for generating agile steering motions for snake-like robots has, nevertheless, not yet been studied extensively, especially when interacting with the environment. The reasons are 2-fold. First, the steering control for snake-like robots is complex, especially when it comes to the sudden change in velocity or direction of travel, as this requires the coordination of bodies with redundant degrees of freedom from one moving pattern to another in a short time. Second, when traditional methods are used on mobile platforms, target tracking is usually divided into tracking and control sub-tasks, which makes it difficult to tune the pipeline jointly, especially considering the aforementioned motion barrier for snake-like robots. To cope with this hard-to-predict tracking and movement complexity, the RL-based control strategies need to map the visual inputs to the joint space directly, in order to perform the corresponding motions, and must operate with adequately defined reward functions to train a policy successfully. Hence, the design of a target tracking controller for snake-like robots based on RL is challenging.

To design a target tracking controller for snake-like robots, this paper proposes a RL-based strategy. Our main contributions to the literature are summarized as follows. First, we offer a novel alternative to solving the target tracking task for a snake-like robot via reinforcement learning. The learned policy directly transforms the external and internal observations to a sophisticated motion pattern for performing perception-action coupling tasks. It is worth to note that this RL-based method can be applied in different types of snake-like robots instead of designed solely for the one used in this work. Second, we propose a customized reward function that takes contiguous distances into calculation. With this reward design, the learned locomotion successfully solves adaptive target tracking tasks, and, more surprisingly it also learns the ability to keep the target within its visual field, even though this behavior is not specifically rewarded. Third, we propose a tracking accuracy metric that takes both the distance and direction into consideration. Based on this metric, we demonstrate that the learned locomotion outperforms the model-based locomotion in terms of tracking accuracy.

## 2. Related Work

As our work is related to the perception-driven locomotion of snake-like robots and perception-driven algorithms based on reinforcement learning, we briefly review the state-of-the-art research on both aspects in the following.

### 2.1. Vision-Based Snake-Like Locomotion

Trajectory or target tracking of snake-like robots is important and operators usually control their locomotion by indicating the expected direction of its head module (Kamegawa et al., 2004; Fukushima et al., 2012; Yamada et al., 2013; Tanaka and Tanaka, 2015). Under the velocity constraints, which prevent the body from slipping sideways, trajectory tracking locomotion control of snake-like robots has been investigated (Matsuno and Mogi, 2000; Prautsch et al., 2000; Transeth et al., 2007; Ishikawa et al., 2009; Tanaka and Matsuno, 2014). Liljeback proposed a straight line path-following controller of a planar snake-like robot under the Line-of-Sight (LOS) guidance law, but the robot's head could not track the desired trajectory (Liljeback et al., 2011). Matsuno derived a dynamic model to avoid the singular configuration of the robot body. Using this control law, their snake robot can accomplish trajectory tracking of the head module without converging to a singular configuration (Matsuno and Sato, 2005). However, their results were only tested on a sinusoid-like track and this dynamics-based method may not adapt to unknown scenarios with changing dynamics. Similar ideas can also be found in Tanaka et al. (2015) and Huang et al. (2017). Xiao performed autonomous locomotion in a known scene and the positions of the snake-like robot and the obstacles were acquired from external web cameras (Xiao et al., 2015). This method, in fact, is a trade-off idea since they could not use the built-in camera in the snake-like robot due to its undulating locomotion pattern. In fact, there are few research efforts about the onboard vision-based locomotion control of snake-like robots, since the undulation of the body cannot provide a stable base for vision sensors. Bing et al. (2019a) proposed an end-to-end target tracking for snake-like robots based on spiking neural network. However, the network controller only outputs the steering signals and the locomotion itself is further generated with model-based methods. The robot “IRS Souryu” equipped with a ToF camera and 3D range sensors performs real-time localization and mapping tasks in a rubble environment (Ohno et al., 2006). A semi-auto snake-like robot with a B/W camera and a structured light sensor was investigated to perform a localization task of a pole, navigate and then climb it (Ponte et al., 2014). A slithering gait that specially designed for snake-like robots to perform target tracking tasks is introduced in Bing et al. (2017). A detailed survey about perception-driven and obstacle-aided locomotion for snake robots can be found in Sanfilippo et al. (2017).

### 2.2. RL-Based Tracking

As a principle approach to temporal decision-making problems, RL-based approaches have been used for solving visual object tracking tasks that aim at finding the target position in contiguous frames and whereby steering the locomotion of an mobile agent. Gaskett designed a mobile robot that can perform visual servoing and wandering behaviors through a Q-learning method (Gaskett et al., 2000; Garcia-Aracil et al., 2011). The work clearly demonstrated that a direct mapping from image space to actuator command using RL is a promising method. Similar work is also given in Asada et al. (1996), Takahashi et al. (1999), Busquets et al. (2002), and Hafner and Riedmiller (2003). Miljkovic presented a novel intelligent visual servo controller for a robot manipulator using RL (Miljković et al., 2013). Based on their control scheme, the performance of the robot is improved and is able to adapt to the changing environment. In the recent ViZDoom platform for visual reinforcement learning, they provided two basic scenarios: a basic move-and-shoot bot and a maze-navigation bot. Yun developed a reinforcement learning scheme to utilize labeled video sequences for training their action-driven tracker (Yun et al., 2018). In Ding et al. (2018), a partial RL based tracking algorithm was proposed to achieve adaptive control of a wheeled mobile robotic system working in highly complex and unpredictable environment. The controller required less calculation time than other optimization technologies and exhibited higher accuracy at the same time. As far as we are aware, to date, there have been no studies that employ reinforcement algorithm to control snake-like robots for performing vision-based locomotion, except for one of our previous research that used RL to design energy-efficient gaits (Bing et al., 2019b).

## 3. Models and Tasks

This section first introduces the models of the snake-like robot. Then, the target tracking task is presented, together with the target tracking metric for evaluating the performances of different algorithms.

### 3.1. Models

**Figure 1:**
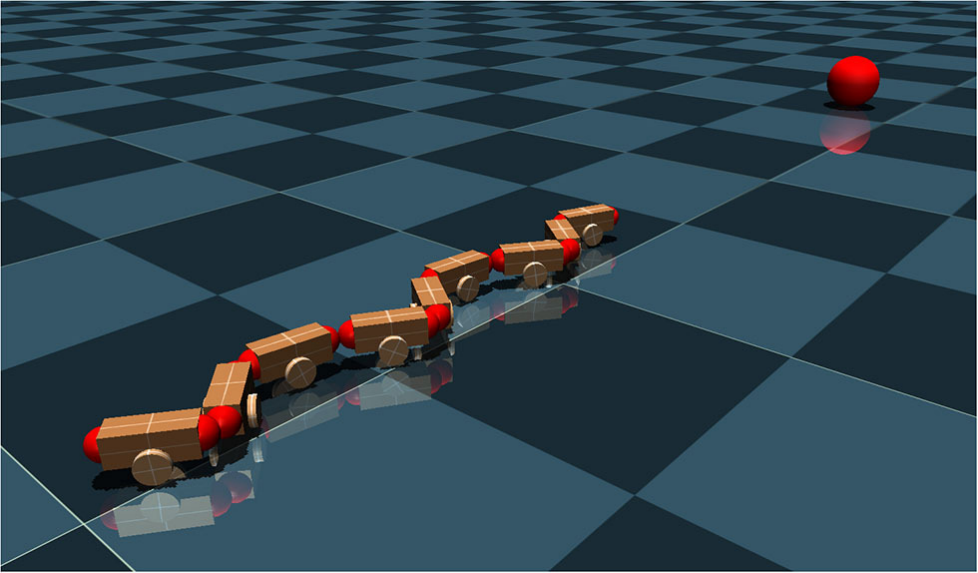
Target tracking scene for a snake-like robot.

The target tracking scene is modeled and simulated in MuJoCo (Todorov et al., 2012), in which a red ball is used as the target and a snake-like robot is the tracker (see Figure 1). The easily detectable sphere has a radius of 0.2*m* and is placed in front of the snake at the distance of 4*m*. The robot is inspired by the *ACM* snake-like robot (Hirose, 1993), which uses eight joints and nine identical modules. A head camera is used as the visual system for the snake-like robot, which is positioned in the center of the first head module. Due to the camera position and view volume, the ground area in front of the robot is clipped during rendering. The purpose of this camera is to obtain information of the moving target. More details about the model of the robot can be found in Bing et al. (2019b).

### 3.2. Tasks Description

In our target tracking task, the snake-like robot has to follow a target while maintaining a certain distance from it. The target moves with a specified velocity *v*_*t*_ = 0.3*m*/*s* on the trajectories of random tracks with constrained conditions. The random-tracks consists of short straight forward sections linked by abrupt random direction changes with angles between −60° and 60°. A random seed is used to generate arbitrarily random tracks during the training process.

**Figure 2:**
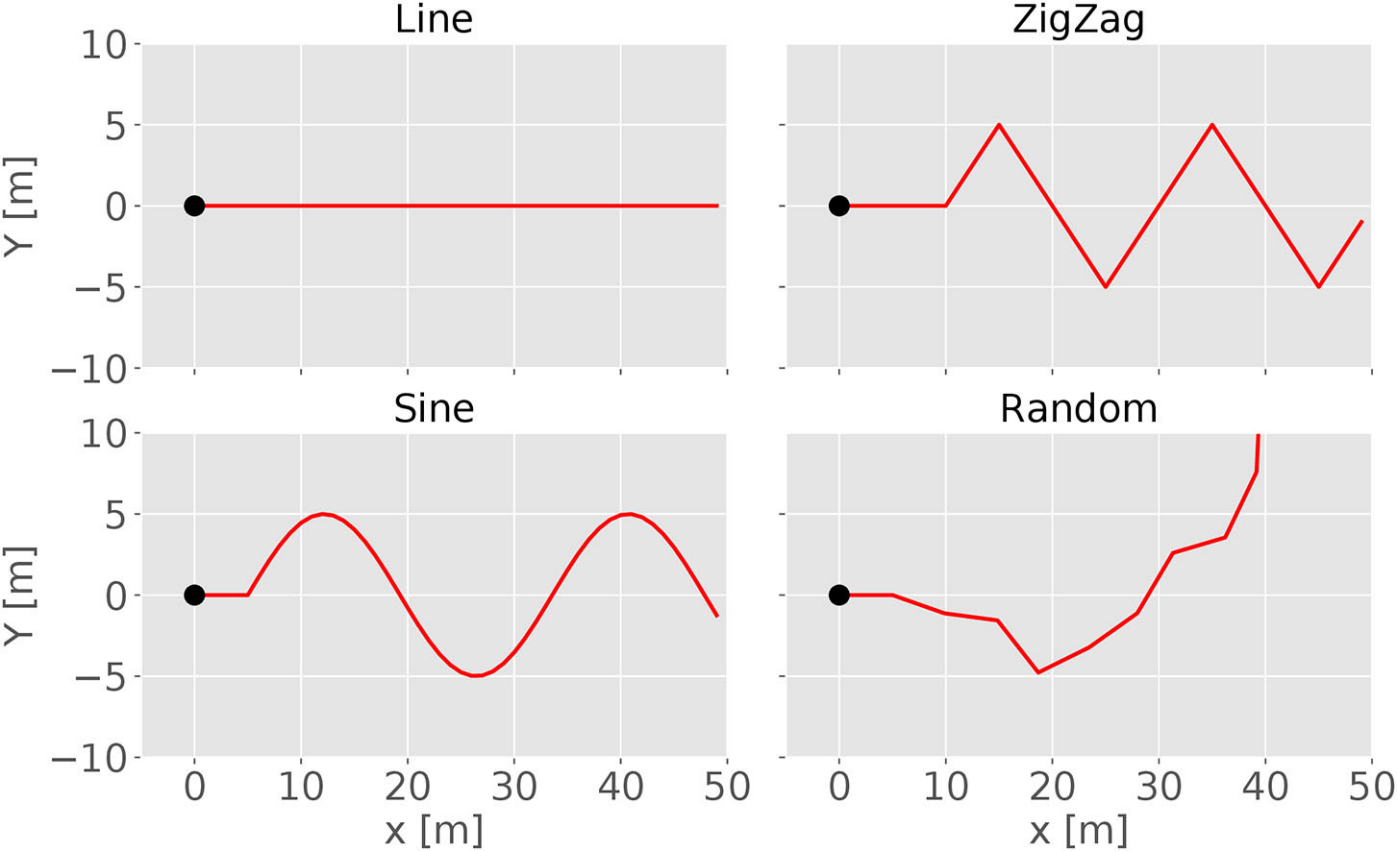
Each of these four diagrams visualizes one of the testing tracks. The tracks define the trajectory of the target. The black dots indicate the start positions of the track. Only a limited length is displayed because the tracks are continuing. The first three figures show the *line, zigzag*, and *sine* track, respectively. The last sub-figure gives an example of a *random* track.

For evaluating the performances of the controller, we also design four predefined tracks for testing as shown in Figure 2. The *line* track is used to test a simple forward locomotion and is therefore the easiest. For testing the steering behavior, the *wave* track offers a continuous curve where the robot has to alternately change its steering direction. A modified sinus wave defines the *wave* track. The *zigzag* and *random* track scenarios test the robot's capability to handle abrupt directional changes. The *zigzag* track is defined by alternating abrupt left and right turns of 60°. The *random* track consists of short straight forward sections linked by abrupt random direction changes with angles between −60° and 60°. Heess et al. (2017) described that starting with easier tasks for training supports a faster learning process in RL. Thus, all the tracks have a short straight segment at the beginning, which enhances the learning process because it is easier to move straight forward at the beginning of a training process.

### 3.3. Tracking Metrics

**Figure 3:**
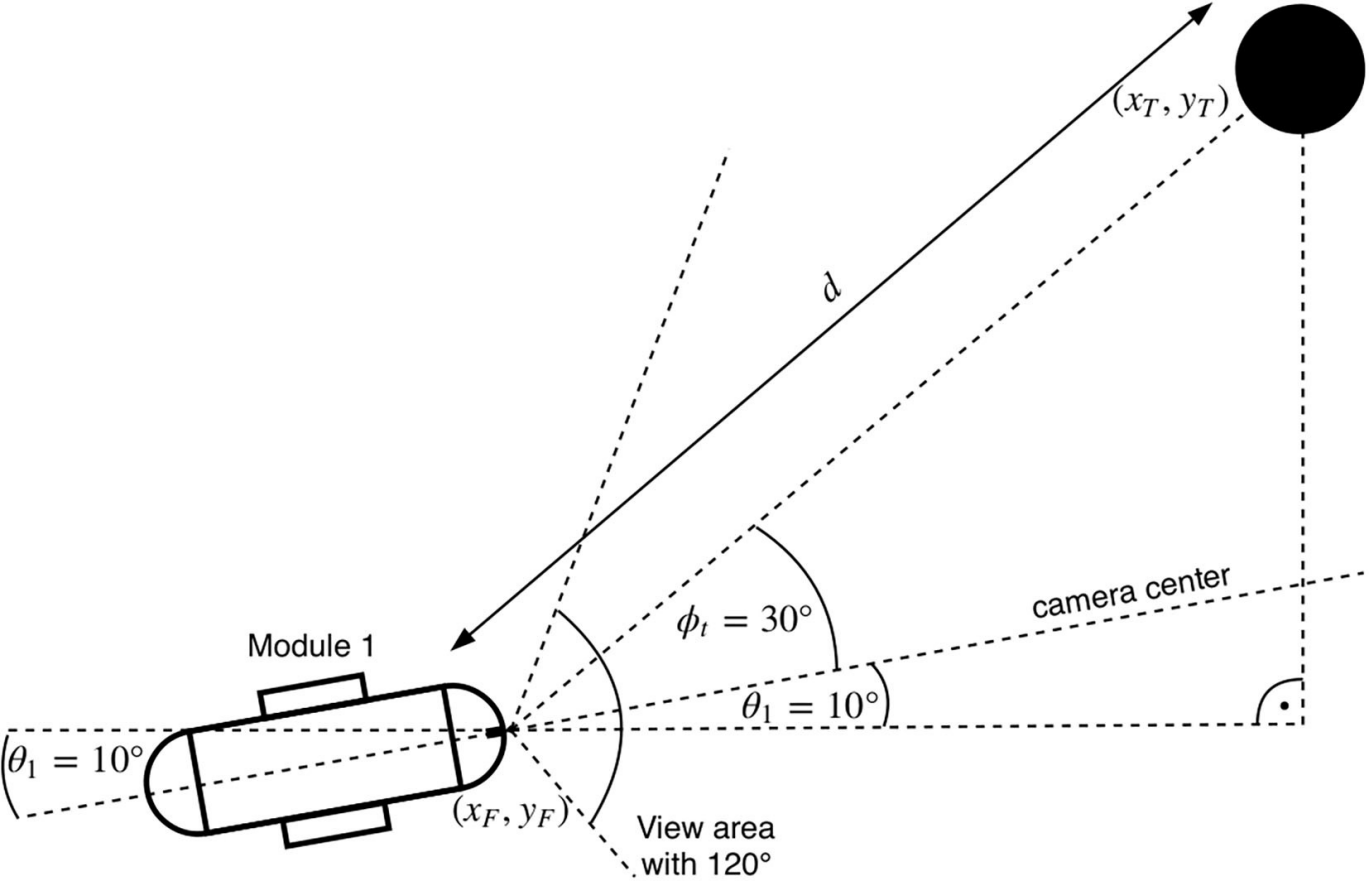
An overview of the used angles between the camera and the target. It shows the first module and the target with their corresponding relative angles from a top view perspective. Here, the head to target angle ϕ_*t*_ has a value of 30°. The global angle of the head θ_*F*_ is 10°. The target position is at (*x*_*T*_, *y*_*T*_) and has a Euclidean distance *d* to the camera position (*x*_*F*_, *y*_*F*_).

As illustrated in Figure 3, the location of the target at time *t* can be represented as **X**_*T*_(*t*) = (*x*_*T*_(*t*), *y*_*T*_(*t*)) in the global frame of reference $R_{G}$. Similarly, let the position of the snake-like robot at time *t* be denoted by **X**_*F*_(*t*) = (*x*_*F*_(*t*), *y*_*F*_(*t*)). For simplicity, we discretize the time with steps of δ_*t*_ = 0.01*ms* and use the notation *n* to refer to the *n*^*th*^ time step. Let *d*(*n*) = ||**X**_*T*_(*n*) − **X**_*F*_(*n*)||_2_ be the distance between the target and the robot at time-slot *n*, where ||·||_2_ denotes the *L*_2_ norm. In addition, $\theta_{T}=\arctan\frac{y_{T}\left(t\right)}{x_{T}\left(t\right)}$ represents the global angle of the target and $\theta_{F}=\arctan\frac{y_{F}\left(t\right)}{x_{F}\left(t\right)}$ is the angle of the head module in $R_{G}$. Then, the absolute relative angle between the head and the target ϕ_*t*_ can be calculated as

(1) $$\begin{array}{r} \phi_{t}=|\theta_{T}-\theta_{F}| \end{array}$$

An expected tracking is to minimize the distance difference from *d*(*n*) to desired distance *d*_*t*_ and aims at the target in the middle of the field of view (FoV) to greatest extent. Thus, the Averaged Tracking Error (*ATE*) is defined as

(2) $$\begin{array}{r} ATE=\overset{N}{\underset{i=0}{\sum }}(\frac{\left|d\left(n\right)-d_{t}\right|}{d_{t}}\times \frac{\phi_{t}\left(n\right)}{\phi_{t_{max}}})\times \frac{1}{N}\text{,} \end{array}$$

where *N* is the amount of the time steps. |*d*(*n*) − *d*_*t*_| calculates the absolute distance error in the normal direction. $\frac{1}{d_{t}}$ is used to calculate the error ratio against the desired target. $\frac{\phi_{t}\left(n\right)}{\phi_{t_{max}}}$ indicates the target's deviation in the FoV, where $\phi_{t_{max}}=60^{∙}$.

It should be noted that the tracking metric *ATE* is not used the reward function for our RL controller, since the reward signal only depicts a desired behavior that keeps a distance with the target while the tracking metric specifically measures the tracking accuracy. For RL tasks, it is more realistic to simply use an intuitive reward instead of regularizing it with a specific metric.

## 4. Baseline Example

This section briefly explains the start-of-the-art and widely used method for controlling the locomotion of snake-like robots, which is the model-based *gait equation* controller.

The *gait equation* method is a kinematic locomotion controller that describes the gaits using a sinusoid-like wave. This method was first proposed as the *serpentine curve* (Hirose, 1993) by Hirose who gained inspiration from real snakes. In this work, an undulation gait equation developed in Tesch et al. (2009) is used for the purpose of comparison. The *gait equation* controller is modeled as

(3) $$\begin{array}{r} \alpha \left(m,t\right)=\left(\frac{m}{M}x+y\right)\times A\times \sin\left(\omega t+\lambda m\right)+C\;\text{.} \end{array}$$

α(*m, t*) presents the joint angle value at time *t*, where *m* is the joint index and *M* is the joint amount. λ and ω are the spatial and temporal frequency of the movement, respectively. The spatial frequency represents the cycle numbers of the wave and the temporal frequency represents the traveling speed of the wave. *A* is the serpentine amplitude and *x* and *y* are the constants for shaping the body curve. *C* is the amplitude bias for steering the direction of a slithering locomotion.

The target tracking locomotion for the *gait equation* controller is divided into two sub-tasks, namely, lateral localization and speed control. Similar ideas can be also found in Bing et al. (2017) and Bing et al. (2019a). In the FoV of the robot, the target will be identified as a group of red pixels. For the lateral localization control, the moment of the red pixels is calculated and then used as the control target for a proportional integral (PI) controller, since it indicates the relative position of the target in the FoV of the robot. For the speed control, the number of the red pixels are counted to represent the distance from the robot to the target. In order to have a more accurate estimation, the visual image is rendered with a higher resolution 128 × 80 × 3. This is because a higher resolution can generate more amounts of red pixels for the same target and then result in a more accurate control performance. Due to the page limit, the implementation details of the *gait equation* controller will not be further explained. To ensure a fair comparison, we make many attempts to find the best control parameters to optimize the performance. But only the best tracking results are selected as the baseline example for further usage.

## 5. Proposed RL-Based Controller

This section presents the details of the proposed RL-based controller, including the construction of the network and the training configuration and results.

### 5.1. Reinforcement Learning Setup

**Figure 4:**
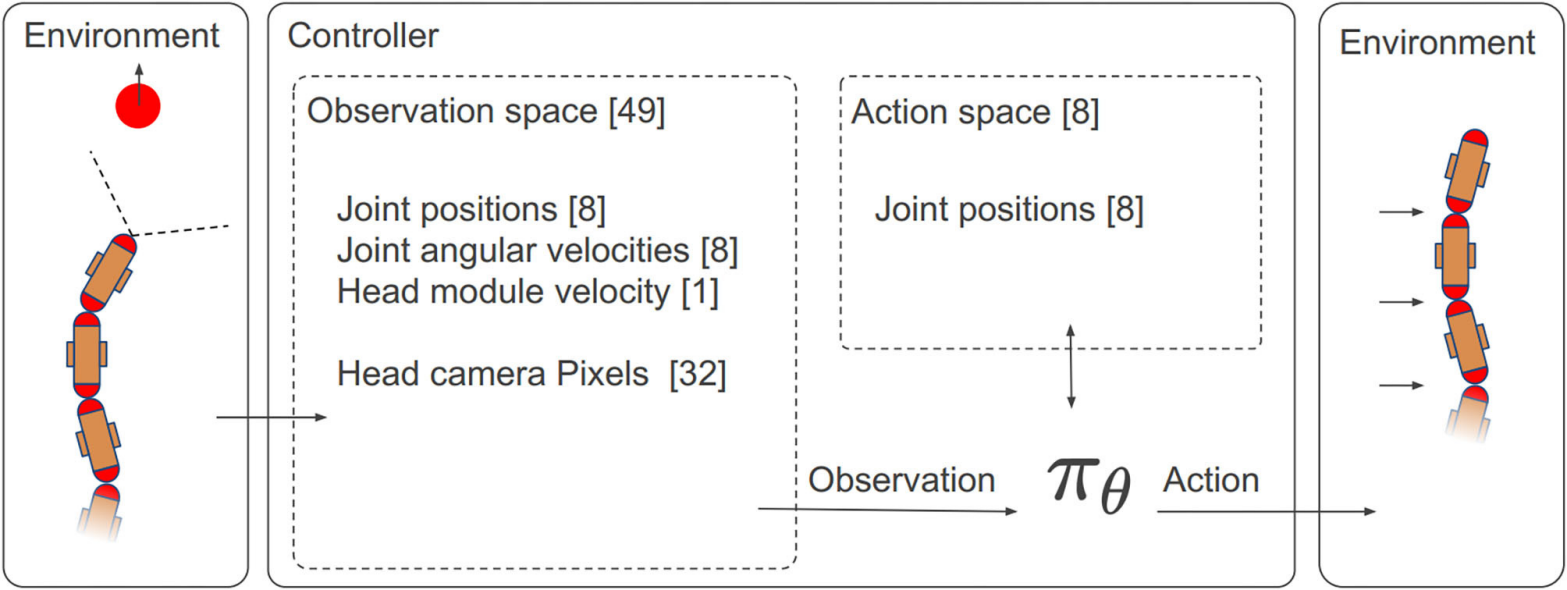
The overall architecture of the RL controller with the details of the observation space and the action space.

The most important components of a RL controller are the observation space, the action space, and the reward function. The overall architecture is shown in Figure 4.

#### 5.1.1. Observation Space

**Figure 5:**
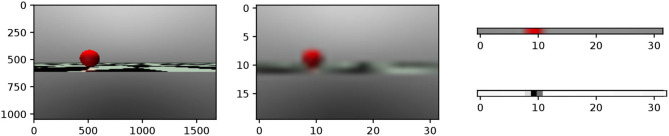
This figure shows the visual information of the target at the distance of 4.0 m with 30° on the left. The left figure is the original image with 1, 685 × 1, 050 from the sensor. The middle figure is rendered with 32 × 20 pixels. The right array of pixels from the 10th row is used to estimate the relative position and distance of the target.

The snake-like robot solely use a RGB vision sensor to track the target. Due to the undulation of the locomotion, the rendered image from the robot keeps shifting in the horizontal direction. In order to reduce unnecessary dimensions and enhance the computing efficiency, the following steps describe the image processing pipeline:

1. The image is directly rendered with 32 × 20 × 3 pixels. The middle figure in Figure 5 shows an example of the rendered RBG image with 32 × 20 × 3 resolution.
2. The 10th row is extracted from the rendered image, since this line contains the pixels at which the target is located, as shown at the top right of Figure 5.
3. The color space is then transformed from RGB to gray with values in the range of [0, 1] based on the intensity of the red pixel, as shown at the bottom right of Figure 5.

The resulting 32 pixels *p*_10,1−32_ contain information about the target's relative position. Furthermore, its distance can also be estimated by the gray value intensities of the target's corresponding pixels. This value increases when the target is closer and decreases when the target is further away. These changes are caused by the illumination of the bright background. The further away the target is, the smaller it is rendered, resulting in a lower pixel count.

The controller also has to control the locomotion of the snake-like robot. To be able to propel forward, it performs a lateral undulation motion pattern with its joints. At each time step, it receives the current joint angular positions α_1−8_ and the angular joint velocities ${\overset{∙}{\alpha }}_{1-8}$ to learn the locomotion and represent the proprioceptive awareness of the robot. In addition to these parameters, the controller also receives the global head module velocity along the moving direction *v*_1_. It has been observed that this helps it to estimate its global velocity. This is required to control the velocity of the robot. In summary, an overall 49-DOF observation space is used in this work, as shown in Table 1.

**Table 1:** The observation space ${\text{o}}_{i}^{t}$ of the controller.

**Symbols**	**Descriptions**
α_1−8_	Relative joint angular positions
${\overset{∙}{\alpha }}_{1-8}$	Relative joint angular velocity
*v*_1_	Absolute head module linear velocity
*p*_10,1−32_	Pixel 1–32 of the 10th row of the camera image

#### 5.1.2. Action Space

The action space ${\text{a}}_{i}^{t}$ of the RL controller corresponds to the eight joint positions of the robot, which linearly map finite continuous values in the range of [−1.5, 1.5] to [−90°, 90°].

#### 5.1.3. Reward Function

In the target tracking task, the snake-like robot follows a moving target while maintaining a specified distance *d*_*t*_ from it. Meanwhile, the robot should also try to maintain the target in the center of its FoV. Therefore, the distance-keeping and lateral localization in the FoV are the two criteria to find a successful behavior.

Thus, a distance-keeping reward is designed. Let *d*_*r*_ = 2*m* define the distance radius from *t*_*d*_ = 4*m* to its maximum distance *d*_*max*_ = 6*m* and minimum distance *d*_*min*_ = 2*m*. In this approach, the reward represents the distance change between the head module and the target. The less distance changed toward the target distance *d*_*t*_, the higher the reward. Similarly, the lower reward is received by increasing or decreasing the distance from the target distance *d*_*t*_. The reward function is defined as follows:

$$\begin{array}{l} r_{d}=\left(1-\frac{\left|d_{t}-d_{\text{after}}\right|}{d_{r}}\right)-\left(1-\frac{\left|d_{t}-d_{\text{before}}\right|}{d_{r}}\right)\\ \;=\frac{\left|d_{t}-d_{\text{before}}|-|d_{t}-d_{\text{after}}\right|}{d_{r}} \end{array}$$

Here, *d*_before_ defines the distance before the action, whereby the distance after the action is defined as *d*_after_. Note that the distance change can also be denoted as velocity, since the measurements are time dependent. The term |*d*_*t*_ − *d*| calculates the absolute distance difference between *d*_*t*_ and the current head position. The resulting normalization $\frac{\left|d_{t}-d\right|}{d_{r}}$ is 0, if *d* = *d*_*t*_ and 1 if *d* = *d*_*t*_ ± *d*_*r*_. This effect is inverted by $1-\frac{\left|d_{t}-d\right|}{d_{r}}$. As a result, the maximum reward of 1 is achieved if *d* = *d*_*t*_.

In the task of autonomous target tracking, it is important to maintain vision of the target in order to react to its movement changes. It is worth noting, different from the evaluation metric defined in (2), that the reward function does not explicitly reward that behavior. The agent has to learn independently that it must observe the target's position in order to follow it.

### 5.2. Training

In order to map the input (observation ${\text{o}}_{i}^{t}$) and the output (action ${\text{a}}_{i}^{t}$), a fully connected 2-hidden-layer neural network is constructed as an approximator to the policy π_θ_. The input layer and the output layer share the same dimensions as the observation space ${\text{o}}_{i}^{t}$ and action space ${\text{a}}_{i}^{t}$. The proximal policy optimization (PPO) algorithm adapted from is used to train the network, since PPO performs better on continuous action space tasks while being much simpler to implement and tune (Schulman et al., 2017). We train our policy network on a computer with an i7-9750H CPU and a Nvidia RTX 2070 GPU.

**Figure 6:**
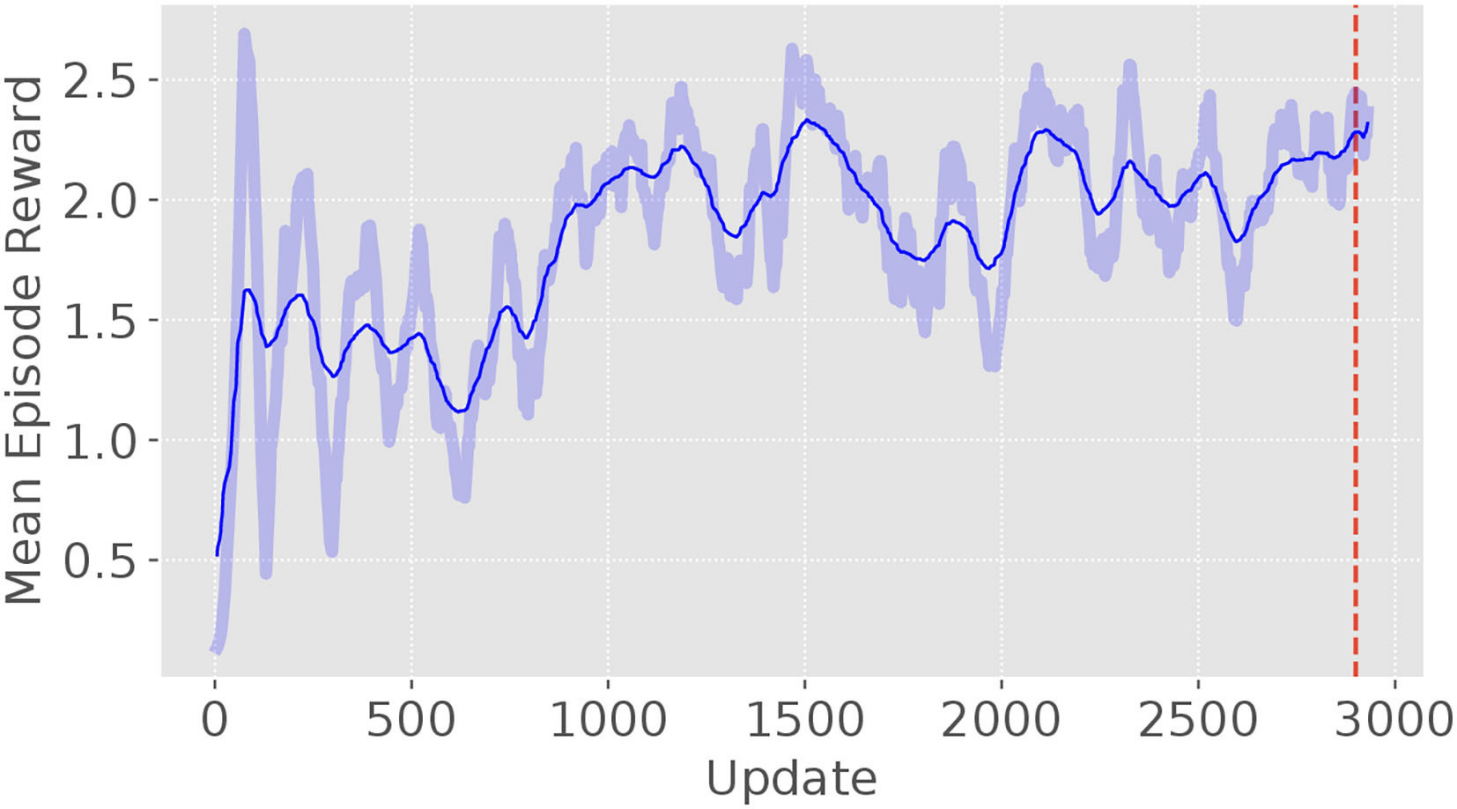
The learning curve of the autonomous target tracking model. It is trained with 3 million time steps with 1,000 time steps per episode and 1,024 time steps per update.

The model is trained by using the random track with a changing random seed for every episode. Therefore, a variety of tracks are generated and the model will not overfit to a specific track. This is necessary because it has been observed that the model tended to overfit while training on unvarying trajectories. As result for overfitting, the controller was not be able to adapt to other trajectory patterns. Based on the learning curve, a total of 3 million time steps (about 3,000 updates) were used for training (see Figure 6). The training process will terminate itself either when the target is out of view or reaching the end of the total time-steps. The mean reward gradually increases and levels up at around 2.4 with some fluctuation. This is because performance of the controller varies from the randomly changing track for each episode. The model at update 2,900 was selected for the further usage.

## 6. Results and Discussions

This section will first describe the performance of the gaits generated by the RL controller in testing tracks. Then, we compare our gaits to the gaits generated from traditional model-based method in terms of tracking accuracy. Finally, we will give the limitation of the simulated results.

### 6.1. Results

**Figure 7:**
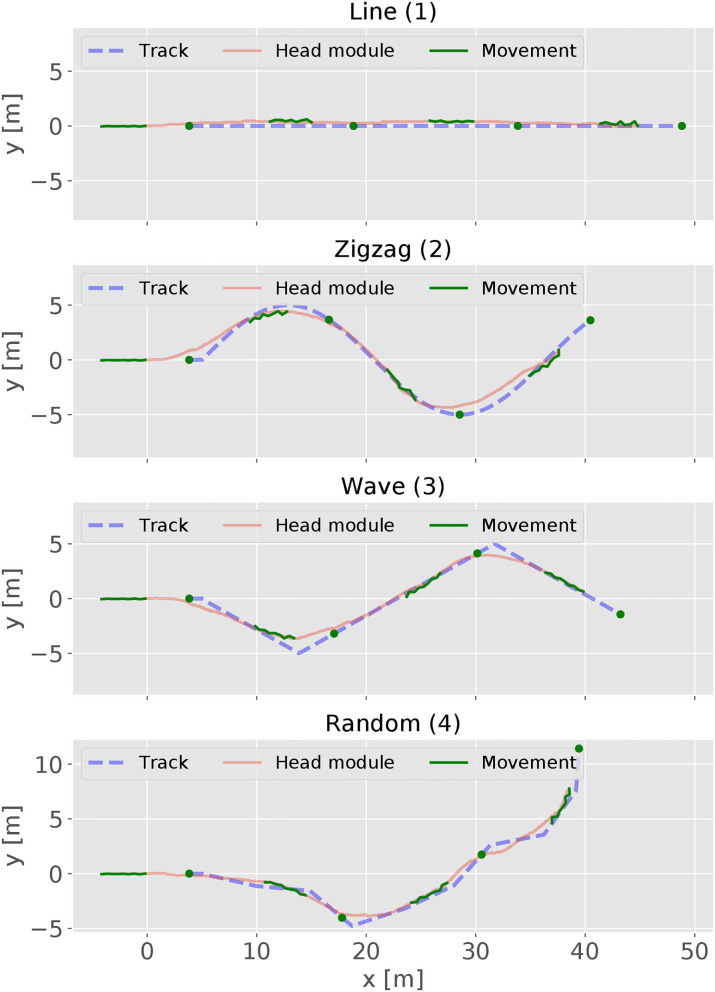
The trajectories of the snake-like robot and the testing tracks. In addition, the body curve of the snake-like robot and the target position are added and captured every 1,000 time step.

**Figure 8:**
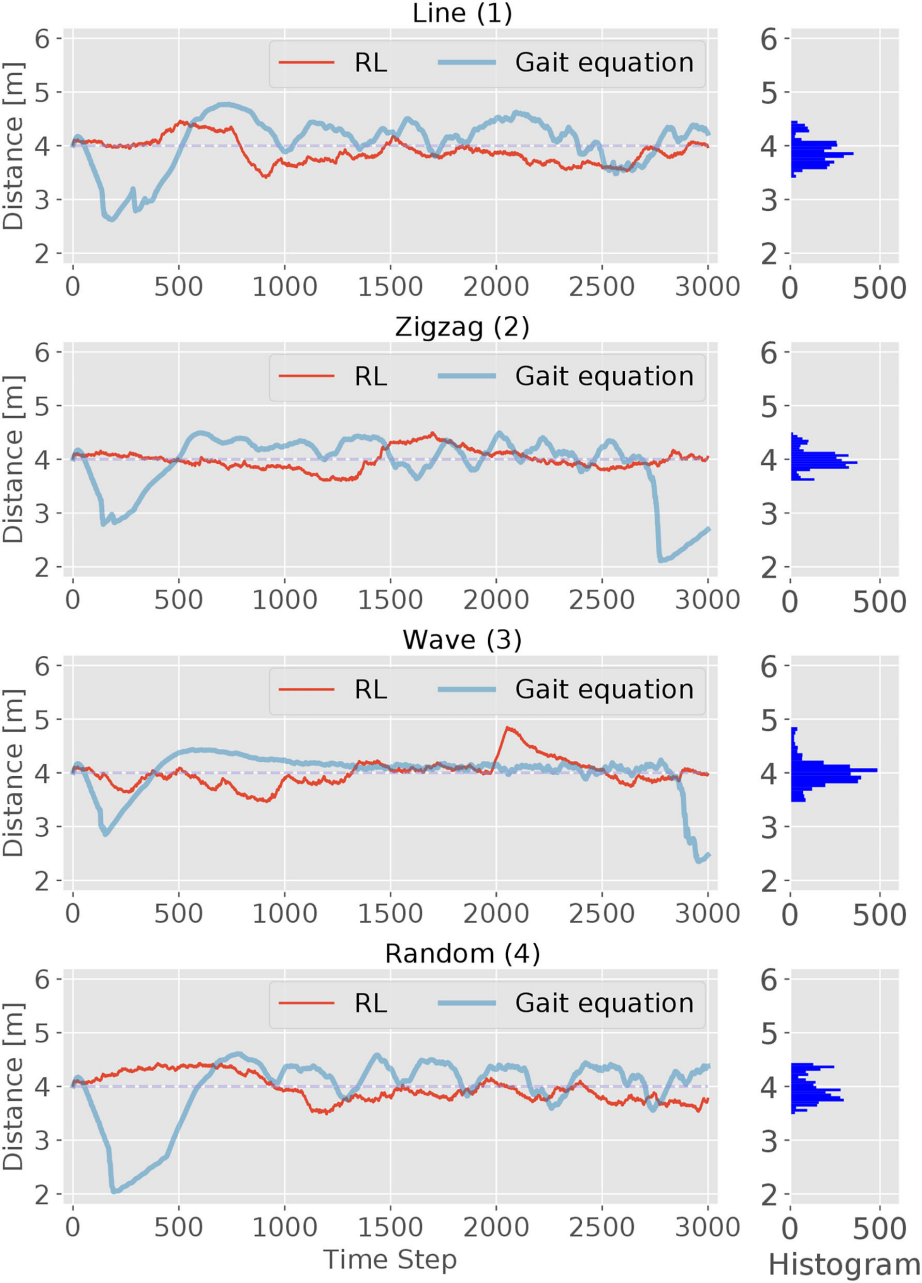
The performance of the RL and *gait equation* controller in maintaining a certain distance from the target is shown in this figure. Each diagram represents one episode run on a track with 3,000 time steps. The target distance is set to 4.0 m and the range limits are set at 2.0 and 6.0 m. The histogram on the right of each diagram shows the distance distribution of the RL controller. Overall the distance varies in an adequate range around the target distance.

The performance of the RL controller was tested on four different tracks (see Figure 2). For evaluation, the episode length is set to 3, 000 time steps. The trajectories of the head module of robot during the evaluation are shown using red solid lines in Figure 7, together with its corresponding track pattern (blue dash lines). In addition, the body curves are plotted every 1, 000 time step with the target position at that time using green lines and dots. For all four tracks, the RL controller was able to successfully follow the target. By comparing the trace of the snake-like robot and the track, a variation is observed in which the trajectories are not matched to each other. In some sections, they go in parallel or cross each other. This indicates that the snake-like robot is not heading directly for the target's position. In some cases, the head module's trace takes a shortcut in the curves of the target's track. However, the trace of the head module is maintaining a visible minimum distance. We can thus conclude that the controller performs a successful path-following behavior. Besides, the controller had to maintain a certain distance to the target. The red lines and the histogram in Figure 8 show the distance distribution of runs on all tracks. In all runs, the distance varies around the target of 4.0 m which is measured at the center of the head. The oscillation movement of the head causes constant minimal distance changes. Table 2 shows the statistics of the runs. As result, the controller was able to maintain the distance from the target with an adequate variance.

**Table 2:** The statistics for the head to target distances (unit: meters).

**Track**	**Mean**	**Std**	**Min**	**Max**
*Line*	3.99	0.18	3.60	4.49
*Random*	3.96	0.24	3.48	4.43
*Wave*	3.99	0.24	3.46	4.85
*Zigzag*	3.88	0.22	3.40	4.46

### 6.2. Comparisons

In order to evaluate the performances of the RL controller against the model-based method, the *gait equation* controller (section 4) was also executed on those four testing tracks.

We first compare the performance of the tracking accuracy. Figure 8 shows the traces of the distance between the target and the robot for both controllers. In general, we can observe that the RL controller has a better tracking accuracy than the *gait equation* controller. For the RL controller, it exhibits better performances at the beginning process on all different tracks and keeps the distance very close to the desired value. Then with the accumulated error, all these four figures reach a relatively large error at some point, but then correct its direction to the right course. For the RL controller, the lag of tracking is much more smaller. After the starting of the movement, RL-based controller also exhibits better tracking accuracy. For the *gait equation* controller, it deviates most at the beginning of the movement for all four tracks. This is because the controller needs to response to the changing visual information and the effect will only show after the error has been accumulated for a period of time.

**Figure 9:**
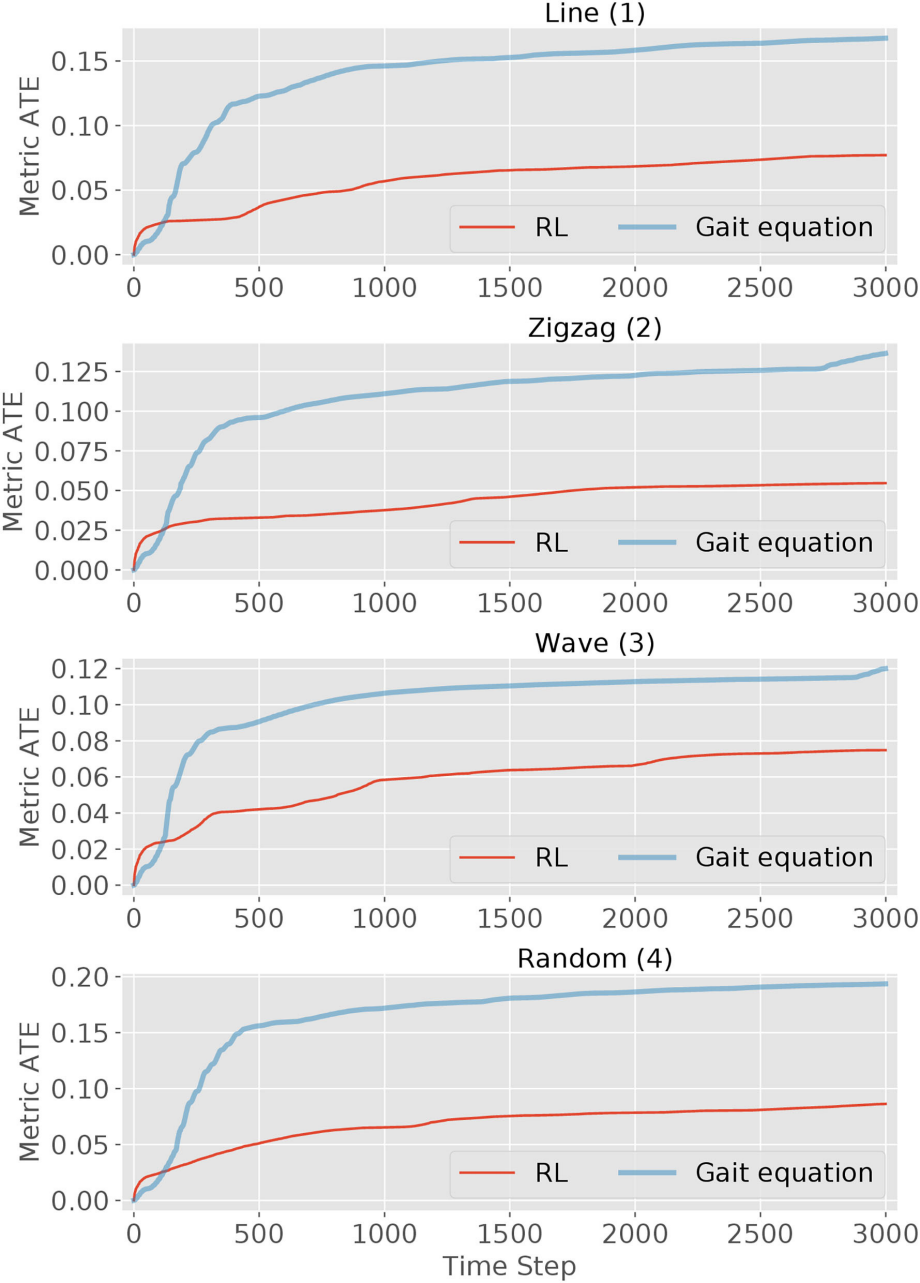
The tracking metric curves of the RL controller and *gait equation* controller over the elapsed time (3,000 timestep in total) in four scenes. The tracking metric is defined in (2).

The second performance indicator is the tracking metric *ATE* defined in (2). The *ATE* is plotted over time steps in Figure 9. It can be observed that for both controllers, the metric curve gets higher due to the accumulated error with time passing by. But for all four scenes, the RL controller outperforms the *gait equation* controller: by around 50% in the simple *line* and *wave* scene and by 70% in the other difficult scenes. In conclusion, the RL controller outperform the *gait equation* controller both in terms of distance tracking accuracy and the averaged tracking error.

Since our training will terminate itself once the robot lose sight of the target, there will be no target recovery behavior obtained during training, such as retaining. In fact, the target recovery behavior is associated with some memory-like function that can predict the motion tendency of a moving object.

### 6.3. Limitations

It is worth noting that, our RL-based controller is demonstrated by simulations now and has not been support with results of physical snake-like robot yet. In order to ensure the validity of the simulated results, we first try to close the simulation-to-reality gap by setting simulation parameters with real-world properties (e.g., dimension, density, friction, etc.). Second, all the methods implemented in simulation can also be produced in a real-world setup.

For prototype experiments, the main challenge is how to train the RL controller in a real-world setup, which usually requires millions of episodes. Different from a robotic arm that can be set to its initial condition easily, there is no good way to reset the training scene for mobile robots in real world. Some algorithms (Fu et al., 2017; Hwangbo et al., 2019) may directly transfer the learned policy from simulation and implement it in real-world scenario. But this is out of the scope of this paper.

## 7. Conclusion

Performing target-tracking tasks for snake-like robots is a challenging task, since it not only involves designing agile locomotion patterns for the robot, but also overcoming difficulties to obtain stable visual information due to the inherent undulatory motions. In this paper, we try to solve this complex perception-to-action control task by using reinforcement learning, which directly maps the vision space to the joint space and reduces the computational complexity of dealing with object tracking and robot motion control in separate components. In our test scenarios, the learned gait shows much better tracking performances than the model-based method. Our work contributes to designing sophisticated and efficient moving patterns for perception-driven tasks with a snake-like robot.

Our future work will aim at performing tracking tasks with more complex visual information. For instance, the perception of the visual information can be replaced with more sophisticated technologies. To improve the adaptability of our RL controller, we will further investigate locomotion skills for more challenging scenarios, such as in a obstacle surrounding environment and the capability to recover tracking when the target runs out of the visual field of the robot.

## Data Availability Statement

The original contributions presented in the study are included in the article/supplementary material, further inquiries can be directed to the corresponding author/s.

## Author Contributions

ZB, CL, and AK brought up the core concept. ZB and CL conducted the experiments and analyzed the results. ZB, CL, ZJ, LC, and KH wrote the paper. All authors contributed to the article and approved the submitted version.

## Conflict of Interest

The authors declare that the research was conducted in the absence of any commercial or financial relationships that could be construed as a potential conflict of interest.
